# Refixation of a Large Osteochondral Fragment with Magnesium Compression Screws—A Case Report

**DOI:** 10.3390/life13051179

**Published:** 2023-05-12

**Authors:** Adrian Deichsel, Lucas Klaus Palma Kries, Michael J. Raschke, Christian Peez, Thorben Briese, Johannes Glasbrenner, Elmar Herbst, Christoph Kittl

**Affiliations:** Department of Trauma, Hand and Reconstructive Surgery, Albert-Schweitzer-Campus 1, University Hospital Muenster, Building W1, 48149 Münster, Germany; adrian.deichsel@ukmuenster.de (A.D.);

**Keywords:** osteochondrosis dissecans, OCD, magnesium compression screw, magnesium implant, osteochondral refixation

## Abstract

Introduction: Osteochondrosis dissecans (OCD) is a disease affecting the subchondral bone and the overlying articular cartilage. The etiology is most likely a combination of biological and mechanical factors. The incidence is highest in children >12 years old and it predominantly affects the knee. In high-grade OCD lesions, free osteochondral fragments usually are refixed via titanium screws or biodegradable screws or pins. In this case, headless compression screws made from magnesium were used for refixation. Case report: A thirteen-year-old female patient with a two-year history of knee pain was diagnosed with an OCD lesion of the medial femoral condyle. After initial conservative treatment, displacement of the osteochondral fragment occurred. Refixation was performed using two headless magnesium compression screws. At the 6 months follow up, the patient was pain free, and the fragment showed progressive healing while the implants were biodegrading. Discussion: Existing implants for refixation of OCD lesions either require subsequent removal or show less stability and possible inflammatory reactions. The new generation of magnesium screws used in this case did not lead to a gas release, as described for previous magnesium implants, while maintaining stability during continuous biodegradation. Conclusions: The data available to date on magnesium implants for the treatment of OCD are promising. However, the evidence on the magnesium implants in refixation surgery of OCD lesions is still limited. Further research needs to be conducted to provide data on outcomes and possible complications.

## 1. Introduction

Osteochondrosis dissecans (OCD) is an illness primarily affecting the subchondral bone and its overlying cartilage with the risk of disruption of the articular surface, resulting in premature osteoarthritis. Current studies indicate a multifactorial genesis of OCD lesions, although the exact pathogenesis remains unclear. Animal studies showed that premature interruption of the epiphyseal cartilage blood vessels leads to necrosis of the epiphyseal cartilage. These necrotic areas result in failure of endochondral ossification, providing less stability to the overlying articular cartilage. Those unstable regions are more vulnerable to single or repeated mechanical trauma, possibly creating osteochondral lesions [1,2,3]. These results correlate to MRI studies of human pediatric knees where increased vascular regression at typical OCD sites was found [4]. Other studies propose biomechanical factors to be the cause for the development of OCD. Athletic activity was shown to be strongly associated with the development of OCD [5]. Increased nonphysiologic loading of the joint surface, as in coronal malalignment, or after meniscal damage, has also been associated with the development of OCD, supporting the biomechanical theory of OCD development [6,7]. Lastly, increased rates of OCD were observed in the course of various hereditary diseases, making genetic predispositions another explanation for the development of OCD [8,9]. A distinction can be made between the adult and juvenile forms of OCD, with skeletal age being the determining factor. Adult OCD is a rare disease with an overall incidence rate of 3.42 per 100,000 person-years [10]. The incidence rate of the juvenile form is substantially higher. It peaks around 12 years of age, with boys more commonly affected than girls. With an incidence rate of 9.5–29 per 100,000, the knee, predominantly affected at the medial femoral condyle, is the most involved joint, followed by 2.2 per 100,000 elbows, and 2–4.6 per 100,000 ankles [11,12]. While low-grade OCD lesions can be managed nonoperatively with partial weight bearing and activity modification for limited amounts of time, high-grade lesions with free osteochondral fragments should be surgically addressed [13,14]. For the refixation of osteochondral fragments, multiple implants are available, including titanium screws, andbiodegradable implants, such as pins or screws [14]. The main disadvantage of titanium implants is the necessity for implant removal [15]. On the other hand, biodegradable implants made from biopolymer materials might suffer from insufficient fixation strength and higher failure rates [16,17]. A possible alternative might be implants made from magnesium, as magnesium shows favorable mechanical properties, as well as osteoinductive potential, while simultaneously being biodegradable [18,19,20,21,22]. This report describes a case in which a large osteochondral fragment was refixed by the use of two cannulated headless compression screws made from magnesium, leading to a satisfactory clinical result.

## 2. Case Report

**Figure 1:**
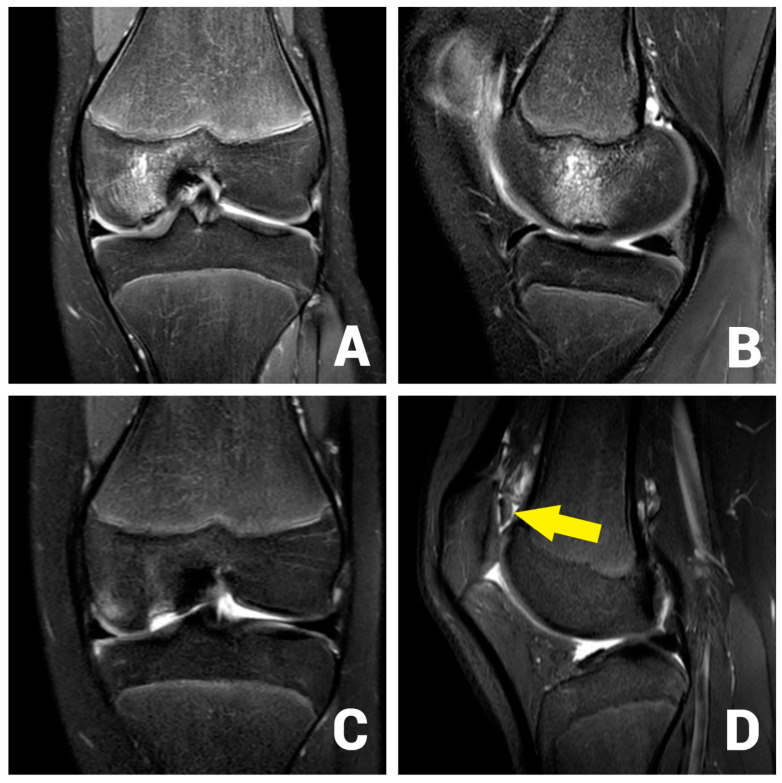
OCD I°–II° of the medial femoral condyle at the typical location in the initial coronar (**A**) and sagittal (**B**) MRI images. After 3 months, the MRI shows an osteochondral defect (**C**) and the displaced osteochondral fragment in the suprapatellar recess (**D**, arrow).

A 13-year-old female patient with a 2-year history of left knee pain was referred to our clinic by a rheumatologic pediatrist. Prior to the consultation, she received treatment for juvenile idiopathic arthritis with uveitis, consisting of methotrexate 10 mg once a week and Adalimumab 20 mg every three weeks. There were no indications of a hereditary burden of osteochondral diseases. In terms of sporting activity, the patient regularly played soccer and swam in a non-competitive manner. On clinical examination, the patient showed a straight leg axis, no effusion, and a range of motion (ROM) of extension/flexion of 0°–0°–130°. On the opposite side, a range of motion of 10°–0°–160° was observed. Hypermobility was also seen in the patient’s other joints (overall Beighton score of 6). The medial and lateral collateral ligaments were stable in 0° and 20° flexion. The Lachman test and the anterior and posterior drawer tests were negative. No indicator of injury to the medial and lateral meniscus could be found clinically. The patella was central and stable, and the apprehension test and J-sign were negative. At rest, the patient felt no pain. However, already at light exertion, knee pain was indicated. The knee pain was initially rated 2 out of 10 on the visual analog scale (VAS) and located on the medial side of the knee. The MRI scan showed an OCD (Clanton type I-II) of approximately 12 × 12 mm at the medial femoral condyle (Figure 1A,B) [23]. The cartilage appeared intact, and no loose bodies could be detected. The remaining cartilage, as well as the cruciate and collateral ligaments, were found to be intact.

After initial conservative treatment with periodical MRI-controls for three months, the patient experienced an aggravation of pain and reported episodes of joint blockage. Apart from a mild effusion, the clinical examination was identical to the previous findings. The joint blockage phenomenon could not be provoked. The MRI scan showed a displacement of the OCD fragment to the suprapatellar recess (Clanton type IV, Figure 1C,D). Due to the young patient’s age, it was decided to refixate the large osteochondral fragment using headless magnesium compression screws.

**Figure 2:**
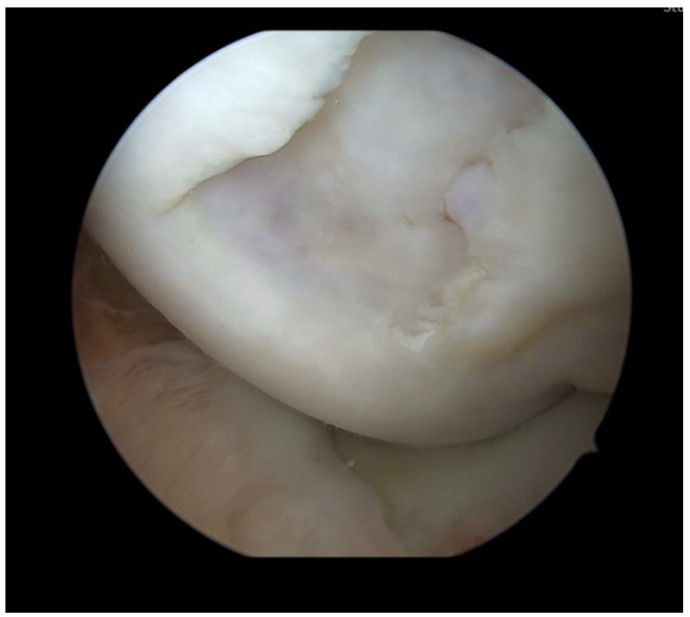
Arthroscopy with inspection of the osteochondral defect in the medial femoral condyle. The defect had a size of 15 × 15 mm and was rated Outerbridge Grade 4.

Surgery was performed with the patient placed in a supine position. A pneumatic tourniquet was put on the ipsilateral thigh and inflated to 100 mm Hg above the systolic blood pressure. Knee testing under general anesthesia showed a mild effusion, full range of motion, and no ligamentous instabilities. A knee arthroscopy was performed using a standard high anterolateral and anteromedial portal. In the medial compartment, the 15 × 15 mm osteochondral lesion (Outerbridge grade 4) at the medial femoral condyle (Figure 2) was observed. Femoral and tibial cartilage conditions were otherwise unremarkable. The medial recess was free and showed no pathologies. The lateral compartment also showed excellent cartilaginous conditions and an unremarkable lateral recess. The menisci and cruciate ligaments were stable under palpation. Since the free osteochondral fragment could not be found in the anterior region of the knee joint, the posteromedial recess and the posterolateral recess were inspected. In the posterolateral recess, the displaced OCD fragment was found and extracted from the knee using a posteromedial portal.

**Figure 3:**
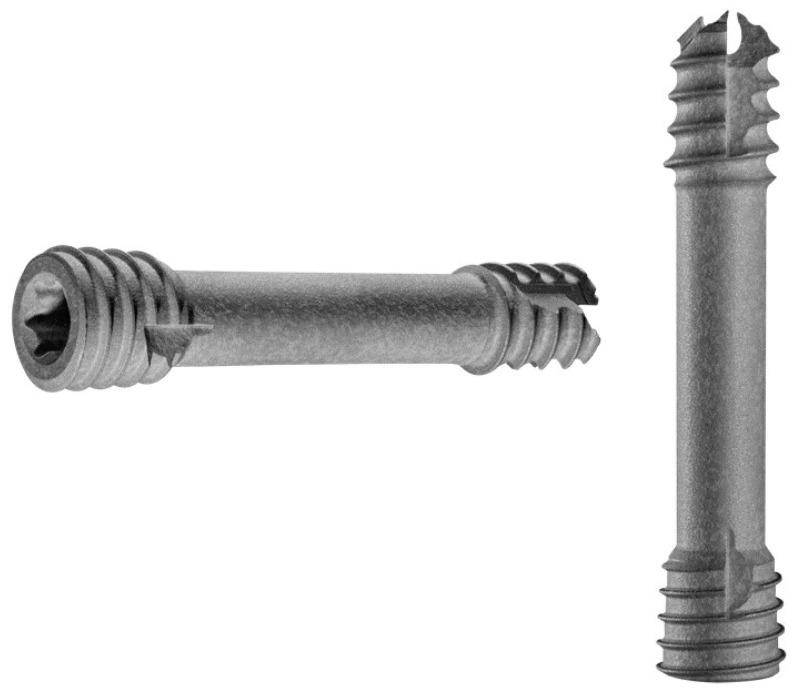
The headless compression screws (Medical Magnesium GmbH, Aachen, Germany) used during refixation of the OCD fragment. © Medical Magnesium GmbH.

**Figure 4:**
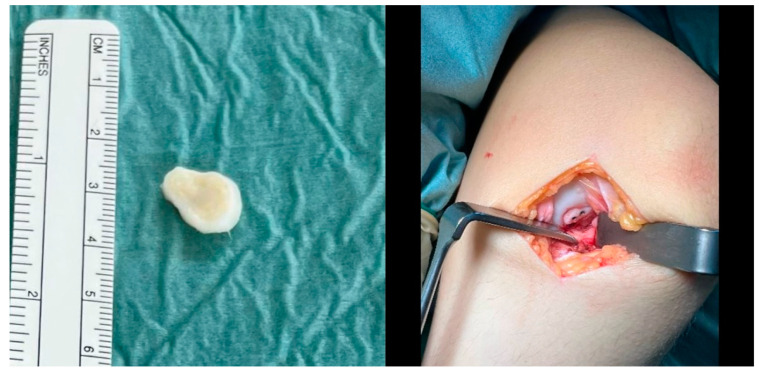
The osteochondral fragment had a size of 15 × 15 mm (**left**) and was fixated with two 2.8 × 20 mm magnesium screws after microfracturing of the osteochondral defect via a medial mini-arthrotomy (**right**).

The fragment was then reduced in size and debrided at the edges (Figure 3). The anteromedial portal was then extended to a medial mini-open arthrotomy. After identifying the osteochondral lesion, a curettage and microfracturing of the lesion with a 1.3 mm K-wire was performed. Subsequently, the OCD fragment was temporarily fixated by two 1.0 mm K-wires, which were subsequently overdrilled using a 2.2 mm cannulated drill. Two cannulated 2.8 × 20 mm headless magnesium compression screws (Medical Magnesium GmbH, Aachen, Germany) were inserted, and the border of the fragment was covered with fibrin glue (Figure 4).

After surgery, the knee was immobilized in a straight knee splint for one week. After one week the active range of motion was limited to extension/flexion of 0°–0°–60°, after 3 weeks postoperatively to 0°–0°–90° and after 5 weeks to 0°–0°–110° to reduce the load on the osteochondral fragment and promote healing. Seven weeks after surgery, the mobile brace was removed. Continuous passive motion was started directly after surgery. For the first six weeks postoperatively, only partial weight-bearing with 10 kg on the injured leg was allowed.

**Figure 5:**
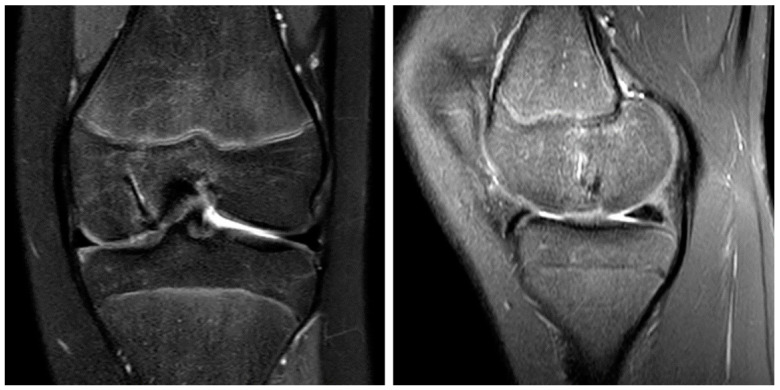
The MRI 6 months after surgery shows the OCD fragment in place and the remodeling process of the magnesium screws in the coronar (**left**) and sagittal plane (**right**).

Seven weeks after surgery, the patient was presented for a scheduled examination. In the clinical examination, no joint effusion, and slight knee extension deficit of 5° with free flexion movement were observed. The knee continued to show ligamentous stability. The reported VAS-Score was 1 out of 10. Six months after surgery, the patient again reported pain in the affected knee (VAS-Score 3 of 10), especially after physical exertion. In the meantime, the patient resumed her previous athletic activities (swimming, soccer). Pressure pain could be elicited over the medial joint space and medial femoral condyle. The MRI scan showed the remodeling of the magnesium screws and the OCD fragment in place without dislocation (Figure 5). We recommended analgetic medication as well as activity reduction (no contact sports) for one month.

**Figure 6:**
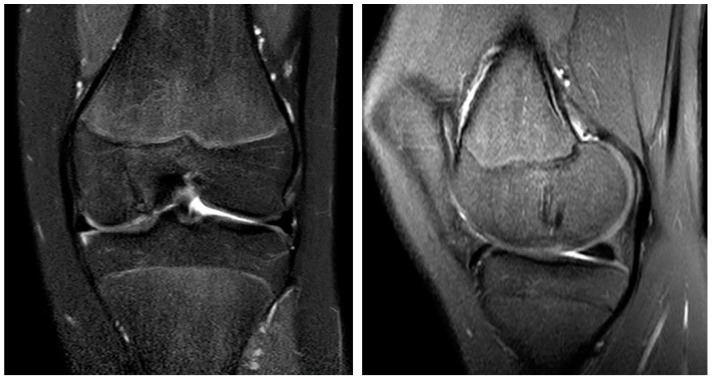
The MRI 10 months after surgery shows the almost entirely healed bone and the continuing remodeling process of the magnesium screws in the coronar (**left**) and sagittal plane (**right**).

At the final visit after 10 months, the patient was free of pain (VAS-Score 0 of 10) and had a free range of motion (10°–0°–160°), equivalent to the uninjured side. The pressure—pain over the medial femoral condyle, which could be provoked in the preliminary examination, was no longer observable. The MRI scan showed progressive healing of the fragment and continuing degradation of the magnesium screws, without apparent formation of gas caverns around the implant (Figure 6).

We finished the therapy with a satisfactory result. Further treatment was taken over by colleagues from pediatric rheumatology, where the patient was previously treated. There, the patient appeared pain-free (VAS-Score 0 of 10) and without movement restrictions 14 months after the operation.

## 3. Discussion

As stated in the introduction, it is difficult to attribute the development of OCD to one factor. Risk factors, in this case, were juvenile idiopathic arthritis, young patient age, and mechanical stress during sports activities. A retrospective study could show an increased prevalence of OCD in patients with juvenile idiopathic arthritis. [24] In cases of OCD with dislocated osteochondral fragments, surgery with refixation of the fragment is the treatment of choice, leading to satisfactory results in a majority of patients [25]. The most commonly used implants for this are cannulated titanium screws, which allow reliable fixation and compression of the fragment to the bone, leading to favorable results [26]. However, when metal screws are used, subsequent arthroscopy has to be performed, typically after six to twelve weeks, when healing has occurred, to remove the implants. Furthermore, protruding screw heads can damage the opposing cartilage, leading to additional lesions [15]. An alternative to metal implants is biodegradable implants (pins, nails and screws), produced from a variety of materials, mostly biopolymers [14]. The advantages of biodegradable implants are the smaller diameter, leading to less cartilage damage during implantation and that implant removal is not necessary. However, the compression strength of these pins and nails is questioned [15,16], and inflammatory reactions to biopolymer materials are reported [27]. In total, a non-union rate of up to 30 %, is described when biopolymer implants are used for fixation of osteochondral fragments [16,17].

Thus, a possible alternative for biopolymer implants may be magnesium as a material for biodegradable implants. Magnesium displays mechanical properties comparable to human cortical bone, which are significantly stronger than most biopolymers available [18,19]. Additionally, magnesium displays an osteoinductive effect, improving both bone growth and fracture healing, in vitro and in small animal models [20,21,22], Marukawa et al. showed that, in a canine fracture model, poly-l-lactide screws were broken 4 weeks after implantation while magnesium screws were not [28]. Windhagen et al. showed that magnesium screws were able to obtain an equivalent postoperative outcome in comparison to titanium screws when used in hallux valgus surgery [29]. However, despite these advantages, magnesium implants suffer from certain drawbacks, which derive from the degradation by corrosion, when in contact with H_2_O, a process during which hydrogen gas is released [30]. Previous generation of magnesium implants could degrade faster than the occurrence of bony healing, leading to preterm failure of the fixation [30,31]. Furthermore, the released hydrogen gas could lead to the formation of gas caverns surrounding the implant [32,33,34]. For the newer-generation magnesium implants, alloying or surface modifications were introduced to reduce these problems.

The screws used in this study are manufactured from a WE43 alloy and plasma electrolytic oxidation (PEO) as a surface treatment was applied. WE43 alloy is a slow-rate corroding alloy with the PEO creating a thin, porous layer for corrosion protection and thereby again slowing down implant corrosion. [35] This was shown to improve the degradation kinetics, allowing bone healing and absorption of the hydrogen gas before the formation of gas caverns. The exact composition, details of surface treatment or time needed for biodegradation of the implants used in this case are not publicly available. During the follow-up of the presented patient, no significant gas formation was observed in the MRI around the used implant and successful healing of the osteochondral fragment was obtained, without the need for implant removal. The postoperative MRI was performed after 6 months. Possibly, hydrogen gas formation might have been visible at an earlier timepoint, however, without a negative influence on healing of the fragment.

In the present case, curettage of the sclerotic ground of the OCD lesion was performed to stimulate healing [36]. Furthermore, microfracturing or microdrilling the bone bed below the fragment, as performed, is recommended, as it is hypothesized that pluripotent mesenchymal stem cells from the bone marrow stimulate healing of the fixed fragment [13]. However, micro fracturing can also lead to the formation of intralesional osteophytes, possibly leading to secondary damage to the cartilage [37,38]. The formation of intralesional osteophytes could not be detected in the present case. Furthermore, fibrin glue was applied, as we feel like this increases the smoothness of the cartilage surface and thereby reduces the friction of the fixed fragment with the opposing tibial side.

Overall, the reported outcomes of unstable OCD fragments tend to be good [39,40,41]. However, there is limited evidence regarding the outcome of magnesium implants for OCD refixation. A case series with 19 patients reported on the use of magnesium pins for refixation of unstable OCD lesions in the knee and the tibiotalar joint [42]. After 1 year of follow-up, 12 OCD lesions completely healed, while revision surgery was necessary in one patient, due to implant failure. Another case report described the case of a refixation of an osteochondral fragment in a comminuted elbow fracture with a magnesium compression screw, reporting a good clinical outcome 6 weeks after surgery [43]. The presented case report supports the notion that a good to excellent short-term outcome can be achieved after internal fixation of osteochondral fragments in OCD. Limitations to the use of headless magnesium screws in OCD refixation surgery are the absence of evidence regarding complications and clinical outcome. To the present date, there is no study comparing headless compression screws made of magnesium to metal or polymer screws in OCD lesions of the knee or other joints.

## 4. Conclusions

In conclusion, we used biodegradable headless compression screws made of magnesium to refixate a dislocated osteochondral fragment in a case of juvenile OCD. Postoperative clinical examinations showed excellent function, a rapid return to sports, and ultimately no pain. The postoperative MRI scans showed good healing and progressive biodegradation of the inserted implants.

The implants used in this case may be a viable alternative to the conventional implants made from polymers or metal for fixation of knee OCD lesions. The presented case indicates that modifications to the implants may have reduced or eliminated previous problems with magnesium implants. However, further prospective clinical studies with comparisons of outcomes and possible complications between magnesium implants and conventional implants are needed to evaluate the additional use in refixation surgery of OCD lesions in the knee.

## Data Availability

The data are not publicly available due to privacy of the presented patient.
